# Cardiovascular Remodeling Experienced by Real-World, Unsupervised, Young Novice Marathon Runners

**DOI:** 10.3389/fphys.2020.00232

**Published:** 2020-03-18

**Authors:** Andrew D’Silva, Anish N. Bhuva, Jet van Zalen, Rachel Bastiaenen, Amna Abdel-Gadir, Siana Jones, Niromila Nadarajan, Katia D. Menacho Medina, Yang Ye, Joao Augusto, Thomas A. Treibel, Stefania Rosmini, Manish Ramlall, Paul R. Scully, Camilla Torlasco, James Willis, Gherardo Finocchiaro, Efstathios Papatheodorou, Harshil Dhutia, Della Cole, Irina Chis Ster, Alun D. Hughes, Rajan Sharma, Charlotte Manisty, Guy Lloyd, James C. Moon, Sanjay Sharma

**Affiliations:** ^1^Cardiology Clinical and Academic Group, St George’s, University of London, London, United Kingdom; ^2^Institute for Cardiovascular Science, University College London, London, United Kingdom; ^3^Department of Cardiovascular Imaging, Barts Heart Centre, St Bartholomew’s Hospital, London, United Kingdom; ^4^Department of Cardiology, Guy’s and St Thomas’ NHS Foundation Trust, London, United Kingdom; ^5^Department of Cardiology, Sir Run Run Shaw Hospital, College of Medicine, Zhejiang University, Hangzhou, China; ^6^Department of Cardiovascular, Neural and Metabolic Sciences, Istituto Auxologico Italiano, IRCCS, San Luca Hospital, Milan, Italy; ^7^Department of Cardiology, Royal United Hospitals Bath NHS Foundation Trust, Bath, United Kingdom; ^8^Infection and Immunity Research Institute, St George’s, University of London, London, United Kingdom

**Keywords:** cardiovascular remodeling, athlete’s heart, sports cardiology, endurance exercise, cardiorespiratory fitness, marathon

## Abstract

**Aims:**

Marathon running is a popular ambition in modern societies inclusive of non-athletes. Previous studies have highlighted concerning transient myocardial dysfunction and biomarker release immediately after the race. Whether this method of increasing physical activity is beneficial or harmful remains a matter of debate. We examine in detail the real-world cardiovascular remodeling response following competition in a first marathon.

**Methods:**

Sixty-eight novice marathon runners (36 men and 32 women) aged 30 ± 3 years were investigated 6 months before and 2 weeks after the 2016 London Marathon race in a prospective observational study. Evaluation included electrocardiography, cardiopulmonary exercise testing, echocardiography, and cardiovascular magnetic resonance imaging.

**Results:**

After 17 weeks unsupervised marathon training, runners revealed a symmetrical, eccentric remodeling response with 3–5% increases in left and right ventricular cavity sizes, respectively. Blood pressure (BP) fell by 4/2 mmHg (*P* < 0.01) with reduction in arterial stiffness, despite only 11% demonstrating a clinically meaningful improvement in peak oxygen consumption with an overall non-significant 0.4 ml/min/kg increase in peak oxygen consumption (*P* = 0.14).

**Conclusion:**

In the absence of supervised training, exercise-induced cardiovascular remodeling in real-world novice marathon runners is more modest than previously described and occurs even without improvement in cardiorespiratory fitness. The responses are similar in men and women, who experience a beneficial BP reduction and no evidence of myocardial fibrosis or persistent edema, when achieving average finishing times.

## Introduction

*“If you want to run, run a mile. If you want to experience a different life, run a marathon*” Emil Zátopek, Olympic long-distance runner.

Running a marathon is an increasingly popular personal challenge for many non-athletes, often with the intention of fundraising for good causes. Approximately 349,000 people across Europe and 414,000 people across North America take part in marathon races every year (Andersen, (2014–2017)). The London Marathon is the third largest in the world (Andersen, (2014–2017)) and currently generates over £60 million/year in charity donations (London Marathon sets another record, 2017). London Marathon runners require no prior experience and there is no qualifying time as a barrier to race entry, with the majority taking part as first time marathon runners (Cave and Miller, 2016).

In the 1970s, it was proposed that the type of person capable of completing a marathon might acquire immunity to atherosclerosis (Bassler, 1977, 1978). It has since been made clear that this is not the case and in fact undertaking vigorous physical activity is associated with a transient 5.9 relative risk of myocardial infarction (Mittleman et al., 1993) and 16.9 relative risk of sudden cardiac death (Albert et al., 2000). The absolute risk of sudden cardiac arrest during a marathon is low at 1.01 per 100,000 participants (Kim et al., 2012) and can paradoxically be reduced by greater habitual vigorous exercise (Siscovick et al., 1984; Mittleman et al., 1993; Albert et al., 2000; Chugh and Weiss, 2015). Over the last two decades, multiple studies have highlighted potential cardiovascular dangers of marathon running including transient left and right ventricular dysfunction (Neilan et al., 2006a; La Gerche et al., 2012; Gaudreault et al., 2013), myocardial injury with release of cardiac troponin (Neilan et al., 2006a; Shave et al., 2010; Lara et al., 2019) and for those engaging repeatedly, myocardial fibrosis (Möhlenkamp et al., 2008; Breuckmann et al., 2009; Wilson et al., 2011; Tahir et al., 2018), coronary calcification (Aengevaeren et al., 2017; Merghani et al., 2017), and arrhythmias (Heidbuchel et al., 2003; Mont et al., 2009).

Despite these reported dangers, each year over 400,000 people apply to the ballot hoping to secure a London Marathon place (McGuire, 2018). Large observational data would suggest that for every hour invested in running, there is a return of 7 h longevity (Lee et al., 2017). Some studies show no ceiling of benefit but progressively diminishing returns with increasing volumes of physical activity (Wen et al., 2011; Kyu et al., 2016; Lear et al., 2017), while others describe a reverse J-shaped curve where potential harm emerges at greater than 10-fold the recommended minimum physical activity levels (Lee et al., 2014; Arem et al., 2015; Armstrong et al., 2015; Schnohr et al., 2015).

It remains debatable whether preparation for and participation in a 42-km (26-mile) footrace constitutes a healthy promotion of increased regular physical activity or a potentially cardiotoxic dose of strenuous exercise (Predel, 2014). Previous studies characterizing the remodeling changes associated with a marathon run have been limited by small sample size (Mousavi et al., 2009; Gaudreault et al., 2013; Arbab-Zadeh et al., 2014), exclusion of participants who did not adhere to structured training plans (Zilinski et al., 2015) or included supervised preparatory training for a much longer period than most typical runners would undertake (Arbab-Zadeh et al., 2014). For these reasons, our current knowledge of exercise-induced cardiovascular remodeling resulting from marathon training is somewhat skewed. Given the popularity of marathon running in modern societies, it is valuable for clinicians, runners, and prospective marathon runners to gain greater understanding of the cardiovascular changes resulting from a single marathon in real-world novice runners, for whom this may represent the greatest athletic feat of their lives. Increasing the generalizability of our findings to real-world novice marathon runners, participants were not excluded for not returning training logs or for failing to follow training plans.

The aim of this study was to assess cardiovascular remodeling in detail occurring in real-world, typical novice marathon runners, inclusive of all those finishing the race, without exclusion of those non-adherent to training programs. We sought to recruit a sufficient proportion of men and women to explore gender differences. In recognition that occult atherosclerotic coronary artery disease is an important confounding factor to outcomes of interest, we restricted our study to subjects aged 18–35 years. This group harbors a low prevalence of atherosclerotic disease and greater cardiorespiratory trainability (Ogawa et al., 1992; Green and Crouse, 1993). We hypothesized that real-world cardiovascular remodeling in unsupervised, novice marathon runners would be more modest than previously described work involving supervised marathon training and that similar responses would be seen in men and women.

## Materials and Methods

### Study Design and Study Population

The study was a prospective observational study. Subjects were considered for inclusion if they were aged 18–35 years old and had never run a marathon distance previously. Individuals were excluded if they had pre-existing cardiovascular disease during preliminary investigations or contraindication to cardiac magnetic resonance (CMR). Novice marathon runners within the specified age range, totaling 4,170, were identified through the database records of the organizers (Virgin Money London Marathon) and received notification of the study through a targeted e-mail advertisement 2 weeks after notification of their place in the 2016 London Marathon. The London Marathon is run over a predominantly flat course, through the capital city center around the river Thames, covering 42.2 km (26 miles and 385 yards). The race organizers received 247,069 applicants for ballot places in 2015 with 51,000 places given, culminating in 39,140 marathon finishers in 2016. Interested runners made contact through a call center and those fulfilling inclusion the criteria were subsequently contacted by telephone and appointed to a study day for recruitment. Written consent was obtained from all participants and the National Research Ethics Service; Queen Square, London committee granted ethical approval (15/LO/086). The trial is registered on ClinicalTrials.gov, number NCT02568072.

**FIGURE 1:**
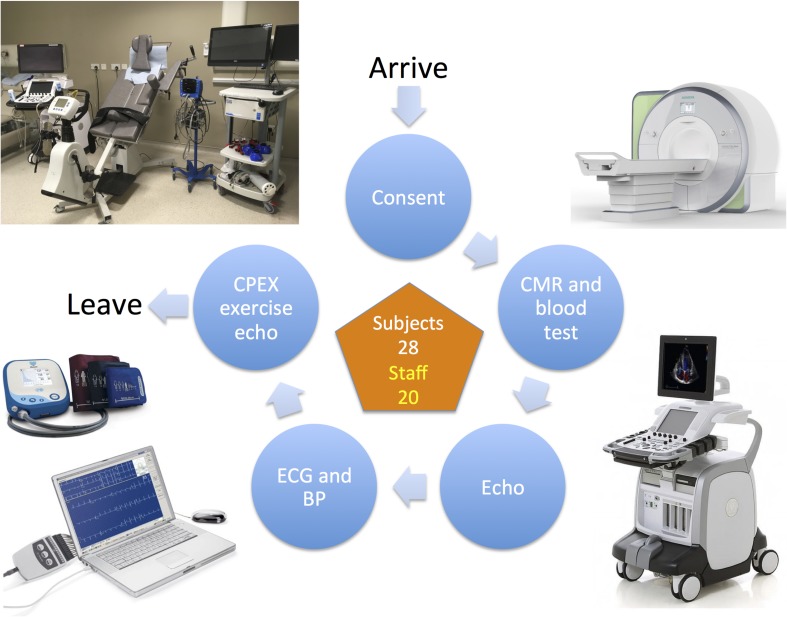
The study visit testing circuit. BP, blood pressure; CMR, cardiac magnetic resonance; CPEX, cardiopulmonary exercise test; ECG, electrocardiogram.

Testing took place in two parallel identical circuits where subjects were changed into gowns (to ensure that no ferromagnetic materials were taken into the CMR environment), height and weight were recorded, followed by cannulation and venipuncture prior to CMR. Subjects then underwent a resting echocardiogram, followed by electrocardiography and blood pressure (BP) measurement. Finally, subjects underwent cardiopulmonary exercise testing using a semi-recumbent tilting cycle ergometer combined with echocardiography in their exercise clothes (Figure 1). Testing was consistent between subjects and between visits.

### Running Training

Subjects were encouraged to follow a beginner’s training plan, consisting of approximately three runs per week, increasing in difficulty over a 17-week period leading up to the London Marathon race, which is the recommendation of the race organizers (London Marathon, 2018) (Beginner 17 Week Training Plan in Supplementary Data Sheet S1). Subjects wishing to follow alternative, higher intensity training plans were not discouraged from doing so.

### Allometry, Bioimpedance, and Blood Pressure

Height was recorded using a standard stadiometer. Weight and body fat percentage were measured using digital bioimpedance scales (BC-418, Tanita, United States). Peripheral and central BPs were measured after 5 min of rest in accordance with international standards (Williams et al., 2004), supra-systolic oscillometric BP was measured in both arms over 10 s at 200 Hz in a semi-supine position using a Cardioscope II BP + device (USCOM, Sydney, NSW, Australia), which employs an upper arm cuff, as previously described (Costello et al., 2015). An ensemble averaged central pressure estimate was derived from the brachial BP and supra-systolic arterial waveforms to estimate central systolic and diastolic BP. At baseline, if the right arm BP was > 10 mmHg greater than the left arm this was used, otherwise the left arm BP was used and repeated measurements of BP used the same arm as the baseline measurement. All BP measurements were recorded by the same investigator (RB) supported by one of five cardiac research nurses working a rotational day schedule, all receiving the same training on recording BP using the Cardioscope II BP + device.

### Electrocardiography

Two-minute ECG recordings were acquired digitally (CardioSoft, GE Healthcare, Milwaukee, WI, United States) according to internationally accepted practices (Eldridge et al., 2014).

### Echocardiography

Resting and exercise two-dimensional echocardiography was performed (Vivid E9, GE Vingmed Ultrasound, Horten, Norway) with standard cardiac views obtained and analyzed according to contemporary European Association of Cardiovascular Imaging guidelines (Lang et al., 2015). Mean frame rates ranged from 72 ± 11 to 78 ± 11 frames/s for long- and short-axis views, respectively. Five cardiac cycles were stored in a cineloop format, optimized for offline 2D speckle-tracking echocardiographic analysis. All automatic image enhancement and harmonics were enabled. Images were saved digitally for subsequent offline analysis by speckle tracking analysis with dedicated software using automated function imaging (EchoPAC Version 113, GE Vingmed Ultrasound AS, Horten, Norway). Twist and torsion were calculated as previously described (Kinova et al., 2018). Studies were performed by nine accredited and experienced cardiac physiologists working a rotational day schedule. Measurements were made by a single investigator (AD) utilizing edge detection software (Auto-EF) for calculation of ventricular volumes and visual confirmation with correction where endocardial border definition was sub-optimal.

### Cardiovascular Magnetic Resonance (CMR)

Cardiac magnetic resonance scans were performed using a 1.5 T magnet (Aera, Siemens Medical Solutions). LV and RV function, volumes, and myocardial mass (excluding papillary muscles) were assessed by cine steady-state free precession sequences and analyzed by a single investigator (AD) using Circle CVI42 (Circle Cardiovascular Imaging Inc., Calgary, Canada) semi-automated software including tissue tracking for strain analysis. Left and right atrial EDV and ESV were derived by manually tracing endocardial atrial contours, as previously described (Petersen et al., 2017). Studies were performed by five experienced radiographers and five experienced clinical research fellows working a rotational day schedule.

Late gadolinium enhancement (LGE) images were obtained 10 min after the intravenous bolus injection of 0.1 mmol/kg gadolinium-based contrast (gadoterate meglumine, Dotarem, Guerbet, LLC).

### Parametric Mapping for Myocardial Tissue Characterization: T1, T2, and ECV

Mid-ventricular short axis pre and post-contrast (15 min post 0.1 mmol/kg Dotarem) T1 maps were acquired by Modified Look-Locker Inversion recovery (MOLLI) sequence [pre: 5s(3s)3s, post: 4s(1s)3s(1s)2s]. MOLLI T1 maps with motion correction were used to generate automated extracellular volume (ECV) maps with contours in the mid-anteroseptum used for analysis, as previously described (Rosmini et al., 2018) based on the following equation:

$$\mathrm{ECV}=\left[1-\mathrm{Hct}\right]\times \left(\frac{\Delta \,\left[1/T\,1\,m\,y\,o\right]}{\Delta \,\left[1/T\,1\,b\,l\,o\,o\,d\right]}\right)$$

Mid-ventricular short axis T2 maps were acquired with the mean segmental pixel value calculated from a region of interest drawn in the mid-anteroseptum.

### Aortic Pulse Wave Velocity

Pulse wave velocity was measured with phase-contrast MR imaging. Phase-contrast sequences were acquired in the ascending aorta (at the level of the pulmonary bifurcation) and the descending thoracic aorta (at the level of the diaphragm) with a prospectively triggered, velocity encoded spoiled gradient echo sequence (flip angle = 20^*o*^; pixel bandwidth = 457 Hz/pixel; uninterpolated resolution = 2.0 × 2.0 mm; acquisition matrix = 192 × 192; echo time = 2.46 ms; repetition time = 9.24 ms, slice thickness 6 mm; FOV: 380 × 380 mm, matrix: VENC: 150 cm/s). For assessment of the aortic arch length, an oblique-sagittal image of the aorta (candy cane view) was obtained using ECG-gated steady state free precession acquisition with breath hold. Calculation of flow wave transit time, aortic distance, and aortic pulse wave velocity was then undertaken as previously described (Bhuva et al., 2019, 2020).

### Imaging Analysis

All resting imaging studies were analyzed by an accredited, experienced cardiologist (AD), blinded to subject identity and time point; 15 CMR studies were randomly selected and reanalyzed independently by another experienced cardiologist (AB or KM) for assessment of inter-observer variability. Exercise echocardiographic studies were analyzed by an accredited cardiac physiologist (JZ) and aortic pulse wave velocity was analyzed by two investigators (AB and NN).

### Blood Samples

Non-fasting blood samples were collected into standard ethylenediaminetatraacetic acid (EDTA) and serum separating (SST) blood collection tubes during intravenous cannulation prior to CMR. On-site laboratory analysis included complete blood count, used to calculate the ECV and a renal chemistry sample, including creatinine and electrolytes. The remainder of whole blood and serum samples were saved in cryovials and stored at −80°C in refrigerators at St George’s, University of London.

### Cardiopulmonary Exercise Testing

Cardiopulmonary exercise testing was performed using a semi-recumbent tilting cycle ergometer (Schiller ERG 911 BP/LS, Schiller, Switzerland) with an incremental ramp protocol of 15–30 W/min, based on a pre-specified algorithm incorporating subject height and gender (Supplementary Table S1). Subjects were exercised to volitional exhaustion with continuous ECG monitoring. Maximal effort was assessed by the presence of a plateau in oxygen uptake seen in Wasserman Plot panel 3, respiratory exchange ratio (RER) > 1.15 and subject perceived exhaustion, as recognized parameters of assessment of effort (Society, 2003). Achievement of maximal predicted heart rate was a less reliable marker of maximal effort with testing conducted on a semi-recumbent cycle, as compared to a treadmill. Breath-by-breath pulmonary gas exchange and ventilation were continuously measured by metabolic cart (Quark CPET, COSMED, Rome, Italy), as previously described (Sharma et al., 2000). The ventilatory threshold was determined by the V-slope method, where two intersecting lines were drawn using dedicated software (Omnia, COSMED, Rome, Italy) on the VCO_2_ vs VO_2_ Wasserman Plot panel 5. Echocardiography was performed after 5 min exercise in the semi-recumbent position to assess augmentation in LV ejection fraction (EF) and stroke volume. To fully characterize exercise ability and potential using the semi-recumbent ergometer, both maximal (maximal VO_2_ and percentage predicted maximal VO_2_) and submaximal indices [oxygen uptake efficiency slope (OUES)] were assessed. In order to appropriately classify cardiorespiratory trainability by accounting for the random within-individual variation and measurement error, a combination of the technical error of measurement (TEM) and the minimal clinically important difference (MCID) were incorporated, as previously described (Williams et al., 2019). Studies were performed by four experienced cardiac physiologists working a rotational day schedule and analyzed by a single investigator (AD). Target exercise times were 5–12 min, if a subject at baseline exercised for more than 12 min to volitional exhaustion the ramp protocol was increased by 5 W/min on post marathon testing.

### Statistical Analysis

Statistical analyses were performed with R version 3.3.0 (R Project for Statistical Computing). Project data were curated using REDCap data tools hosted at University College London (Harris et al., 2009). Data were tested for normality with the Shapiro–Wilk test and assessed in histograms. Normally distributed data are presented as mean ± standard deviation and skewed data are presented as median with inter-quartile range (IQR). Differences between baseline and post marathon time points were compared using a paired *t*-test, if parametric, or Wilcoxon signed rank test if non-parametric and expressed as mean difference. Differences in paired categorical data were compared using McNemar’s test. Comparisons of two unpaired groups (likely responder and likely adverse responder) were assessed using a two-sample independent *t*-test. Comparisons of three unpaired groups (final cohort, injured, lost to follow up) were assessed by one-way ANOVA if continuous or by Chi-squared test if categorical. Reproducibility of measurements both between and within raters was assessed with two-way, mixed single measures intraclass correlation coefficient (ICC) analysis for absolute agreement. ICC > 0.75 = excellent, 0.6–0.74 = good, 0.4–0.59 = fair, and < 0.4 = poor, according to a previously published scale (Cicchetti, 1994). Statistical significance was defined as a two-tailed value of *P* < 0.05.

## Results

### Study Cohort Demographics and Race Finishing Times

**FIGURE 2:**
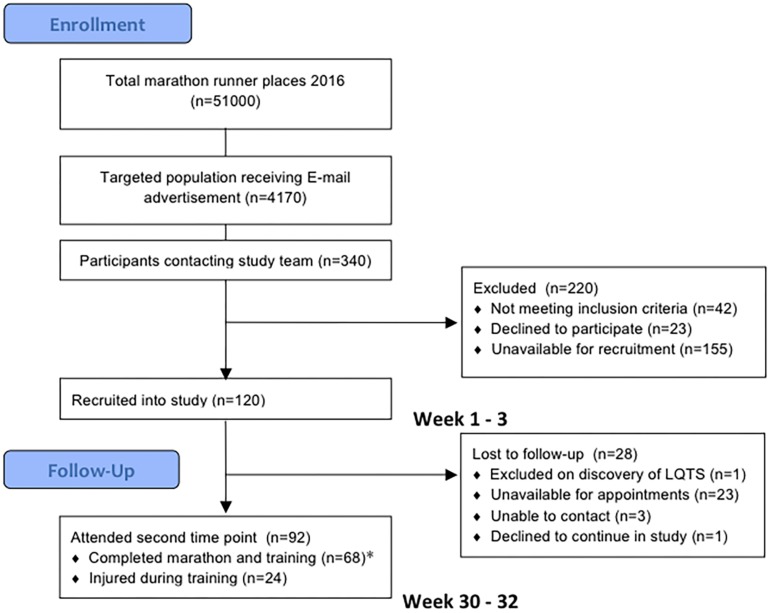
Flow diagram of the trial profile. LQTS, long QT syndrome.

One hundred and twenty subjects were recruited into the study. Twenty-eight were lost to follow up as they only attended the baseline evaluation and did not return for follow up evaluation post marathon, predominantly due to scheduling difficulties in the required timeframe. Of these 28, 12 ran the London Marathon and 16 did not. All 28 were able to confirm that they were alive at the end of the study period and had suffered no clinical cardiac events. In addition, 24 were unable to complete their training due to musculoskeletal injury, they deferred their marathon places and despite not running the marathon they returned for repeat evaluation. These subjects reported continuing light exercise training once their injuries improved but were no longer adherent to a marathon running training plan. They were not included in the primary analysis but their results are appended separately in Supplementary Table S2. The final cohort of marathon completers consisted of 68 novice runners who underwent evaluations at study entry, 186 ± 4 days before the London Marathon in October 2015 and 16 ± 4 days after in May 2016 (Figure 2). Only the results from these marathon completers were included in the main analysis. One marathon completer omitted CMR on post marathon evaluation due to early pregnancy. Baseline measures are presented in Table 1. There was no difference in baseline characteristics between participants completing the study and those who were lost to follow up or injured. Subjects self-reported a median of 2.0 h of exercise per week (range 0–10 h, IQR 1.5–2.5 h) at the time of study entry.

**TABLE 1:** Baseline characteristics of study participants in the final cohort and comparison to subjects not completing training due to injury and lost to follow up.

	Final cohort completing marathon (*n* = 68)	Injured—unable to complete training (*n* = 24)	Lost to follow up (*n* = 28)	*P*-value
Age	29.5 ± 3.2	28.8 ± 3.3	27.9 ± 3.8	0.12
Male *n*(%)	36 (53)	10 (42)	14 (50)	0.64
Ethnicity (%)				0.64
White European	90	96	89	
Other	10	4	11	
Smoking status (%)				
Never smoker	82	83	71	0.65
Ex-smoker	12	8	21	
Current smoker	6	8	7	
Hours of exercise/week	2 [1.5, 2.5]	2 [1.5, 2.6]	2 [1.5, 3.1]	0.89
Weight (kg)	71.3 ± 12.5	72.7 ± 12.6	71.6 ± 14.7	0.74
BMI (kg/m^2^)	23.4 ± 2.9	24.0 ± 3.1	24.4 ± 3.7	0.33
Peak VO_2_ (ml/kg/min)	37.1 [32.8, 42.3]	37.2 [34.0, 40.5]	35.0 [30.4, 40.8]	0.39
Percentage predicted peak VO_2_ (%)	106.7 ± 16.2	111.2 ± 14.9	102.7 ± 17.4	0.18
Systolic BP (mmHg)	119.6 ± 11.8	118.4 ± 11.5	121.8 ± 9.0	0.53
Diastolic BP (mmHg)	73.7 ± 5.3	74.5 ± 5.9	75.6 ± 5.7	0.29
Heart rate (bpm)	66.3 ± 13.8	68.8 ± 14.4	68.4 ± 14.9	0.69
iLV mass (g/m^2^)	64.9 ± 12.1	63.4 ± 9.8	63.2 ± 11.6	0.76
iLV EDV (ml/m^2^)	91.0 ± 14.3	90.0 ± 12.8	90.1 ± 12.0	0.93
iRV EDV (ml/m^2^)	92.6 ± 14.3	93.0 ± 15.4	93.1 ± 13.7	0.99

*Data expressed as mean ± SD if normally distributed. If non-normally distributed data expressed as median [IQR]. BMI, body mass index; BP, blood pressure; EDV, end-diastolic volume; iLV indexed to body surface area left ventricular; iRV, indexed to body surface area right ventricular; VO_2_, oxygen consumption.*

**FIGURE 3:**
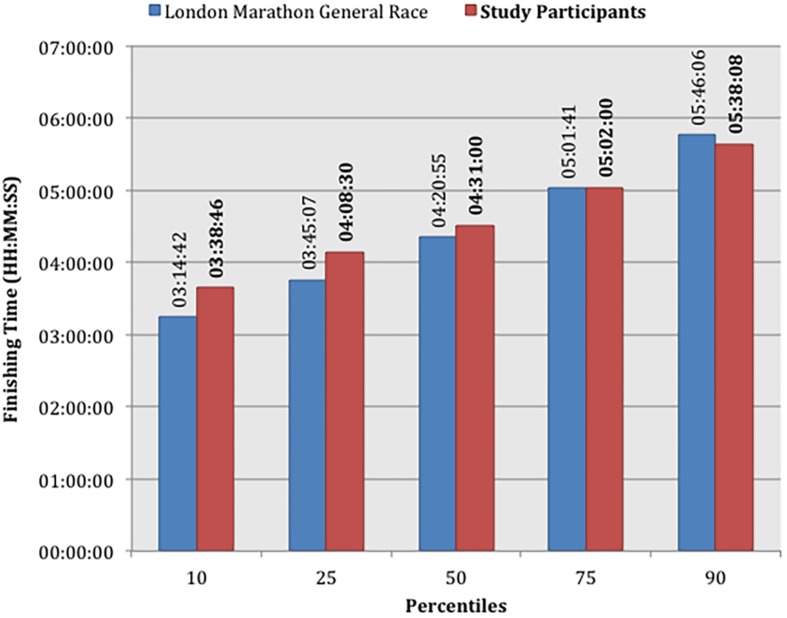
Marathon race finishing times by fastest percentile of each cohort comparing study participants with the London Marathon General Race 2016.

The median race finishing time of the study cohort was 04:31:00 (HH:MM:SS, IQR: 04:08:30–05:02:00, range: 02:56:10–06:51:20). Median finishing times for men and women were 04:14:30 (IQR: 03:42:20–04:42:00) and 04:43:40 (IQR: 04:29:00–05:19:50), respectively. These are above the published median times for the 2016 London Marathon general race, which includes repeat marathon runners, at 04:04:23 for men and 04:39:27 for women (Mirror, 2016; Run247, 2018), though highly comparable (Figure 3).

### Training Data

**FIGURE 4:**
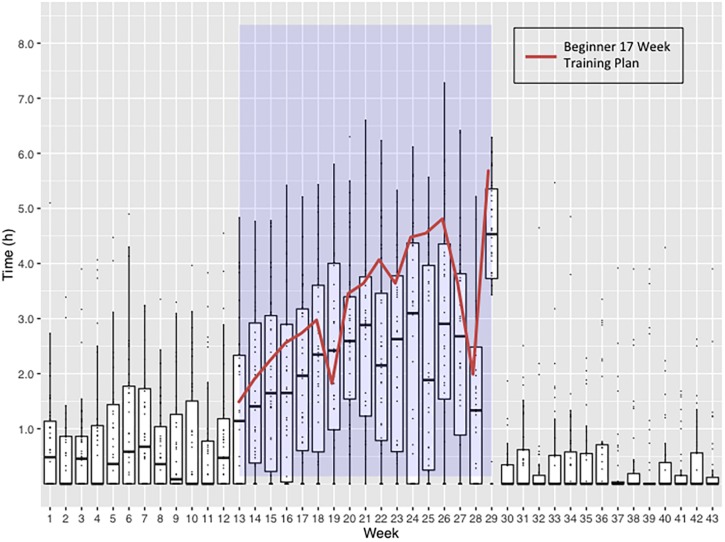
Weekly time spent undertaking exercise by 38 subjects returning training logs. Blue shaded area represents the 17-Week Beginners Training Plan period. Boxplots represent the weekly distribution of time spent in exercise, highlighting the median and interquartile ranges. The Virgin Money London Marathon Beginner 17-Week Training Plan is overlaid to demonstrate the weekly exercise time targets the subjects should have been reaching. Week 17 includes the time spent running the marathon and achieving an average finishing time.

Thirty-eight subjects (32%) provided detailed training and detraining data recorded electronically on portable devices. The training activities of this sub-group are shown in Figure 4 and Supplementary Figures S1, S2. The median training times fell below the recommended 17-week training plan, averaging 78% compliance when studied on a week-to-week basis (Supplementary Table S3).

### Cardiac Structure and Function

Structural and functional CMR measures of cardiac chambers are detailed in Table 2 and Supplementary Table S4, demonstrating balanced eccentric remodeling and no change in LV or RV EF.

**TABLE 2:** Cardiac imaging, hemodynamic, cardiorespiratory, and allometric measurements at baseline and post marathon.

	Baseline	Post marathon	*P*-value
**Echocardiography**			
iLV EDV (ml/m^2^)	58.2 ± 12.2	62.5 ± 13.7	<0.01
iLV ESV (ml/m^2^)	22.4 ± 5.9	26.0 ± 7.0	0.02
LV EF (%)	58.0 ± 4.7	58.5 ± 5.0	0.38
**Exercise echocardiography**			
5-min exercise LV EF (%)	69.0 ± 3.4	66.7 ± 9.1	0.70
5-min exercise iLV SV (ml/m^2^)	81.4 ± 17.4	82.4 ± 19.6	0.20
**CMR and hematocrit**			
iLV EDV (ml/m^2^)	91.2 ± 14.3	94.3 ± 14.8	<0.01
iLV ESV (ml/m^2^)	33.3 ± 7.5	34.8 ± 8.2	0.02
LV EF (%)	63.5 ± 5.0	63.2 ± 5.5	0.71
iLV mass (g/m^2^)	65.2 ± 11.9	68.1 ± 11.4	<0.01
Mean LV wall thickness (mm)	7.0 ± 0.9	7.1 ± 0.9	0.02
Native T1 (ms)	1011 ± 24	1009 ± 36	0.66
ECV (%)	26.8 ± 2.3	25.7 ± 2.4	<0.01
Hematocrit	0.42 ± 0.03	0.43 ± 0.04	<0.01
Native T2 (ms)	45.3 ± 3.5	45.5 ± 3.1	0.76
iRV EDV (ml/m^2^)	92.8 ± 14.4	97.1 ± 16.0	<0.01
iRV ESV (ml/m^2^)	40.5 ± 7.7	42.0 ± 9.1	0.01
RV EF (%)	56.7 ± 4.5	56.9 ± 4.4	0.71
**CPET**			
Peak VO_2_ (ml/min/kg)	37.1 [32.8, 42.7]	37.5 [33.5, 42.0]	0.14
Percentage predicted peak VO_2_ (%)	107.3 ± 16.1	109.6 ± 16.7	0.18
Ventilatory threshold as percentage of peak VO_2_ (%)	61.4 ± 9.6	57.2 ± 8.6	<0.01
Exercise time (s)	578.2 ± 92.3	609.9 ± 101.7	0.01
Peak power (W)	200 [175, 265]	223 [195, 275]	<0.01
OUES (ml/min/L/min)	2686 [2327, 3373]	2582 [2228, 3211]	0.31
Peak HR (bt/min)	170.0 [162.0, 178.0]	171.0 [160.0, 187.0]	0.31
Peak HR percentage predicted (%)	88.67 ± 7.93	87.57 ± 6.26	0.37
Peak RER	1.21 ± 0.09	1.20 ± 0.09	0.67
**Blood pressure and aortic PWV**			
Systolic BP (mmHg)	120 ± 12	116 ± 12	<0.01
Diastolic BP (mmHg)	74 ± 5	72 ± 6	<0.01
CMR whole aorta PWV (m/s)	5.1 [4.8, 5.8]	4.9 [4.6, 5.6]	0.02
**Allometry and renal function**			
Body mass index	23.4 ± 2.9	23.5 ± 2.6	0.42
Body fat (%)	22.7 ± 7.8	22.5 ± 8.6	0.59
Creatinine (μmol/L)	74 ± 14	69 ± 13	<0.01

*Data expressed as mean ± SD if normally distributed. If non-normally distributed data expressed as median [IQR]. BP, blood pressure; CMR, cardiac magnetic resonance; CPET, cardiopulmonary exercise test; ECV, extracellular volume; EDV, end-diastolic volume; EF, ejection fraction; ESV, end-systolic volume; HR, heart rate; iLV indexed left ventricular; iRV, indexed right ventricular; LV, left ventricular; max, maximal; OUES, oxygen uptake efficiency slope; PWV, pulse wave velocity; RER, respiratory exchange ratio; RV, right ventricular; VO_2_, oxygen consumption.*

Cardiac remodeling changes were similar in both men and women, as detailed in Table 3 and Supplementary Table S5. No changes were observed in echocardiographic diastolic function, myocardial strain, peak rotation, twist, or torsion parameters (Supplementary Table S4). LV stroke volume and EF at 5 min of exercise on cardiopulmonary exercise testing did not change after training. Limitations in image quality prevented accurate assessment of echocardiographic indices at peak exercise.

**TABLE 3:** Cardiac imaging, hemodynamic, cardiorespiratory, and allometric measurements at baseline and post marathon, separated by gender.

	Baseline male subjects	Post marathon male subjects	Change	*P*-value	Baseline female subjects	Post marathon female subjects	Change	*P*-value
iLV EDV (ml/m^2^)	98.1 ± 14.2	101.0 ± 14.7	2.9	0.02	83.2 ± 9.5	86.4 ± 10.6	3.2	<0.01
iLV ESV (ml/m^2^)	36.2 ± 8.7	38.2 ± 8.7	2.0	<0.01	30.0 ± 3.7	30.7 ± 5.2	0.7	0.44
LV EF (%)	63.4 ± 5.2	62.3 ± 5.4	-1.0	0.20	63.7 ± 4.8	64.3 ± 5.6	0.6	0.59
iLV Mass (g/m^2^)	72.9 ± 10.4	76.0 ± 8.7	3.1	<0.01	56.3 ± 5.4	59.0 ± 6.3	2.7	<0.01
Mean LV wall size (mm)	7.6 ± 0.7	7.7 ± 0.6	0.1	0.08	6.3 ± 0.6	6.4 ± 0.5	0.1	0.16
Native T1 (ms)	1001 ± 20.20	992.6 ± 30.46	-8	0.18	1023 ± 22.42	1028 ± 33.26	5	0.51
ECV (%)	25.3 ± 1.8	24.3 ± 1.7	-1.0	<0.01	28.3 ± 1.6	27.1 ± 2.3	-1.3	0.02
iRV EDV (ml/m^2^)	100.2 ± 13.9	104.4 ± 15.5	4.2	0.02	84.2 ± 9.3	88.5 ± 12.0	4.3	<0.01
iRV ESV (ml/m^2^)	43.9 ± 7.4	45.9 ± 9.1	2.0	0.05	36.0 ± 5.8	37.6 ± 6.8	1.5	0.11
RV EF (%)	56.2 ± 4.2	56.2 ± 4.5	0	0.98	57.2 ± 4.8	57.7 ± 4.1	0.5	0.63
**CPET**								
Peak VO_2_ (ml/min/kg)	40.5 ± 6.8	42.6 ± 8.0	2.1	0.11	35.2 ± 4.5	35.3 ± 5.9	0.2	0.81
Percentage predicted Peak VO_2_ (%)	99.7 ± 15.8	105 ± 18.0	5.3	0.11	115.2 ± 12.4	117.6 ± 15.1	2.4	0.37
Anaerobic threshold as percentage of Peak VO_2_ (%)	59.6 ± 9.9	55.9 ± 8.4	-3.7	0.05	63.3 ± 9.1	58.6 ± 8.7	-4.7	0.07
**Blood pressure and aortic PWV**								
Systolic BP (mmHg)	124 ± 12	122 ± 11	-2	0.09	114 ± 10	109 ± 8	-5	<0.01
Diastolic BP (mmHg)	75 ± 5	73 ± 6	-2	0.13	73 ± 5	70 ± 5	-3	0.02
CMR whole aorta PWV (m/s)	5.4 ± 1.1	5.2 ± 0.8	-0.2	0.22	5.3 ± 1.0	4.9 ± 0.7	-0.4	0.08
**Allometry and renal function**								
BMI	24.1 ± 3.1	23.9 ± 2.6	-0.2	0.32	22.5 ± 2.4	23.0 ± 2.6	0.4	0.04
Body fat (%)	17.6 ± 5.6	16.4 ± 5.2	-1.2	0.01	28.4 ± 5.7	29.3 ± 6.2	0.9	0.06

*Data expressed as mean ± SD if normally distributed. If non-normally distributed data expressed as median [IQR]. BP, blood pressure; CMR, cardiac magnetic resonance; CPET, cardiopulmonary exercise test; ECV, extracellular volume; EDV, end-diastolic volume; EF, ejection fraction; ESV, end-systolic volume; iLV indexed left ventricular; iRV, indexed right ventricular; LV, left ventricular; max, maximal; METS, metabolic equivalent of task; OUES, oxygen uptake efficiency slope; PWV, pulse wave velocity; RV, right ventricular; VO_2_, oxygen consumption.*

T1 and T2 values from multiparametric mapping did not change. A 1% reduction in ECV was matched by a 1% rise in blood hematocrit with no change in the myocardial partition coefficient, post-contrast T1 myocardial, or blood values. There was no evidence of LGE in any subject, including the additional 24 subjects who did not run the marathon due to injury but attended for re-evaluation.

### Blood Pressure and Arterial Stiffness

Reductions were seen in the aortic pulse wave velocity (ascending to descending aorta), peripheral, and central BP. BP fell by 4/2 mmHg (*P* < 0.01) and aortic pulse wave velocity across the whole aorta fell by 0.2 m/s (*P* = 0.02). In sub-group analysis women, who were also noted to have lower baseline BP, experienced a greater BP reduction than men.

### Cardiopulmonary Exercise Testing

Six (9%) of subjects at baseline exercised to volitional exhaustion in over 12 min, for whom the ramp protocol was increased by 5 W/min when returning for repeat evaluation post marathon. The remaining 62 subjects (91%) were tested on the same ramp protocol post marathon as the baseline exercise tests. Mean baseline exercise time was 09:38 ± 01:32 (MM:SS), which increased to 10:10 ± 01:42 post marathon (*P* = 0.01).

No changes were seen in maximal oxygen consumption (VO_2_), as absolute values and percentage of predicted peak, OUES, or maximum metabolic equivalents achieved (METS), despite a mean increase in exercise time of 32 s and a median increase in peak power achieved of 15 W (*P* < 0.01). The ventilatory anaerobic threshold fell post marathon, both in absolute value and as a percentage of maximal VO_2_.

**FIGURE 5:**
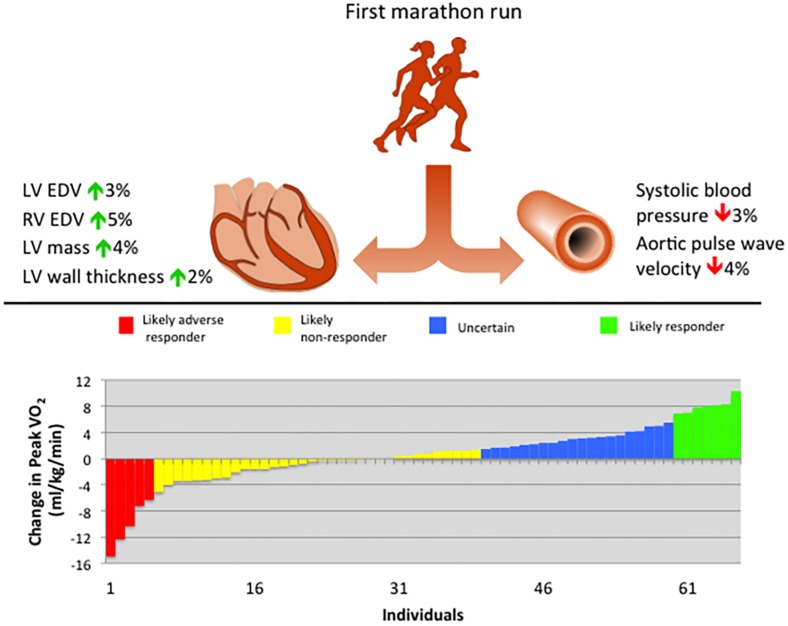
Summary of principal findings of cardiovascular remodeling following training for a first marathon run, including waterfall plot of cardiorespiratory fitness response. EDV, end-diastolic volume; LV, left ventricular; RV, right ventricular; VO_2_, oxygen consumption.

Using the previously established coefficient of variation of 5.6% (Katch et al., 1982), the TEM was calculated by multiplying this value by the mean baseline peak VO_2_, which was 37.91 ml/min/kg in this cohort, therefore TEM = 2.12 ml/kg/min. Applying previously defined criteria for MCID and peak VO_2_ response (Williams et al., 2019), resulted in 7.6% likely adverse responders, 51.5% likely non-responders, 30.3% uncertain, and 10.6% likely responders in this population (Figure 5).

Those subjects who were likely responders based on the change in peak VO2 (*n* = 7) did not demonstrate differences in systolic function, cardiac dimensions, BP, or aortic pulse wave velocity compared with those subjects who were likely adverse responders (*n* = 5). Comparing the available mean weekly exercise volumes over the 17-week training period between likely responders (*n* = 4) with likely adverse responders (*n* = 3), there were no differences (Supplementary Table S6).

### Electrocardiography

No changes were seen in resting heart rate, PR interval, QRS duration, corrected QT interval, or Sokolow–Lyon voltage (S in V_1_ + R in V_5_ or V_6_, depending on the largest values). The prevalence of voltage criteria for ventricular hypertrophy and early repolarization pattern did not change (Supplementary Table S4).

### Allometry, Body Composition, and Renal Function

There were no significant changes in weight, body mass index or percentage body fat over the study period. Serum creatinine decreased by 5 μmol/l (*P* < 0.01) post marathon.

### Reproducibility of Measurements

Intra-observer and inter-observer agreement for all cardiac imaging measurements were excellent (Supplementary Tables S7, S8).

## Discussion

The aim of this study was to examine in detail the cardiovascular remodeling responses in real-world, young, novice marathon runners and explore any differences between men and women. There were a number of key study findings; first, that running a first marathon results in concentric biventricular remodeling that is more modest than previous longitudinal studies of marathon runners (Arbab-Zadeh et al., 2014; Zilinski et al., 2015). Second, a modest BP reduction was seen. Third, these changes occurred without an improvement in peak VO_2_ and finally, responses were similar in men and women.

### Blood Pressure and Renal Biochemistry

The reductions in peripheral and central BP demonstrated in this study were accompanied by a reduction in aortic pulse wave velocity, which is intriguing and suggests that a reduction in vascular stiffness may play a mechanistic role in normotensive, young exercising individuals. These findings have been reported previously in an extended cohort of this study, including older runners and finding greater regional distenibility in the descending aorta after marathon running (Bhuva et al., 2020). Though a 4/2 mmHg BP reduction seems small, this is highly consistent with the effect of exercise on BP reported in large meta-analyses, which is comparable with the effect of antihypertensive medication (Cornelissen and Smart, 2013; Naci et al., 2018) and also on aortic pulse wave velocity (Ashor et al., 2014). In terms of clinical relevance, if sustained, a 2 mmHg systolic BP reduction would be expected to reduce mortality from stroke by 10% and from vascular and ischemic heart disease by 7%, even in a low-risk, normotensive population (Lewington et al., 2002), underscoring the important role of increased physical activity in public health policy. It is recognized that age-matched, premenopausal women have lower BP than men (Maranon and Reckelhoff, 2013). In sub-group analysis women were noted to have a greater BP reduction post marathon than men, however, as we have limited information regarding important confounding factors such as training volume, intensity, lifestyle, and menstrual cycle stage, this finding should be interpreted with caution. Whether a favorable vascular remodeling response might contribute to the markedly lower incidence of sudden cardiac arrest during exercise in women (Marijon et al., 2011) merits further investigation.

A fall in serum creatinine is also an intriguing finding. Though marathon running has been associated with a substantial increase in serum creatinine and renal tubular injury on urine microscopy immediately afterward, this improves after 24 h (Mansour et al., 2017). Regular running training is associated with a fall in serum creatinine 2 weeks before a marathon race (Zilinski et al., 2015) and 2 weeks afterward, despite the acute rise on race day (Hewing et al., 2015). Future studies examining this dynamic relationship between exercise and renal function would be valuable, particularly including assessment of arterial wall mechanics and endothelial function.

### Cardiac Structural Remodeling

We demonstrate a balanced, eccentric remodeling response, which is comparable between men and women. These changes are modest in comparison to previous, smaller studies involving running training in preparation for an endurance event with key differences in study populations and methodologies summarized in Table 4.

**TABLE 4:** Comparative summary of longitudinal cardiac remodeling studies in marathon runners including preparatory training.

Study	Year	Subjects, *n*	Mean age (y)	Female (%)	Exercise exposure	Imaging modality	Peak exercise (h/week)	Increase in peak VO_2_ (%)	Summary
Arbab-Zadeh et al. Circulation	2014	12	29	42	Running for 1 year—supervised	CMR	7–9	17.6	Increased LV mass by 21%, LVEDV by 18%, LV wall thickness by 16%, RV mass by 30%, and RVEDV by 27%. Early concentric LV remodeling then later eccentric remodeling response. RV remodeling was eccentric throughout
Zilinski et al. Circ cardiovasc imaging	2015	45	48	0	Running for 18 weeks—supervised	Echo	4	3.8	Increased LV mass by 14%, LVEDV by 10%, LV wall thickness by 5%, LV length by 5%, RVEDA by 6%, and LAEDV by 11%. Enhanced LV diastolic function
Present study		68	30	47	Running for 17 weeks—unsupervised	Echo CMR	2.7–3.9*	NS	Increased LV mass by 4%, LVEDV by 3%, LV wall thickness by 2%, and RVEDV by 5%. Modest eccentric biventricular remodeling. BP reduced by 4/2 mmHg and aortic PWV by 4%

CMR, cardiac magnetic resonance; Echo; echocardiography; LAEDV, left atrial end-diastolic volume; LV, left ventricular; LVEDV, left ventricular end-diastolic volume; NS, not significant; RVEDA, right ventricular end-diastolic area; RVEDV, right ventricular end-diastolic volume. *2.7 h/week was the median training time over the 8 weeks prior to the race, recorded from 32% of the study cohort. 3.9 h/week was the median training time over the final 8 weeks of the recommended beginner’s training plan, which the study participants were encouraged to follow.

The intensity of peak training prior to the endurance event was greatest in the study by Arbab-Zadeh et al. (2014) where subjects trained for 7–9 h per week in the last 3 months of a year-long supervised program and demonstrated the greatest magnitude of cardiac remodeling and cardiorespiratory fitness response. Zilinski et al. (2015) training a larger population of older, male runners for 4 h per week achieving an average distance of 40 km per week, demonstrated smaller remodeling and cardiorespiratory fitness responses. Our study provided no supervised training intervention and observed in real-world novice marathon runners that cardiac remodeling is even more modest, without cardiorespiratory fitness improvement. This is consistent with a previously proposed schema that with increasing intensity and volume of training, subjects advance through a spectrum of increasing fitness and cardiac remodeling (Beaudry et al., 2016). The finding of a fall in ECV, though a recognized remodeling response in athletes (McDiarmid et al., 2016), is unlikely to represent a genuine change in myocardial structure as it was proportional to the rise in blood hematocrit, without any changes in the constituent myocardial or blood T1 mapping values pre or post contrast. The change in blood hematocrit is likely to be the result of seasonal variation and training effect, which has been previously described (Banfi et al., 2011).

This study found similar proportionate cardiac structural remodeling responses between men and women, who were advised to follow a 17-week beginner’s training plan. A subsequent analysis of the study by Arbab-Zadeh et al. when comparing seven men to five women, found that despite exactly the same training, women experienced a blunting of cardiovascular response with peak VO_2_, LV mass, and mean wall thickness plateauing after only 3 months of training, compared to months 9–12 in men (Howden et al., 2015). The likely reason for the differences observed in these studies relates to the differences in exercise volume, intensity, and duration. This suggests that there may be a dose–response relationship, where a threshold of exercise stimulus must be reached before differential responses in men and women are seen, which may be influenced by body size, sex hormone profile, and hemodynamic response to exercise (Zemva and Rogel, 2001).

### Cardiorespiratory Fitness

It was unexpected to find no difference in peak VO_2_ and only 11% of runners demonstrating a likely cardiorespiratory training response. The observed fall in ventilatory threshold post-marathon, despite longer exercise time and higher peak power achieved may represent overreaching injury, which has previously been recognized in marathon runners (Kasikcioglu et al., 2008; Sierra et al., 2016). Based on the findings of the HERITAGE Family Study, which demonstrated a strong genetic determination of maximal VO_2_, we had anticipated a potential increase of up to 16% in maximal VO_2_, with wide variability in training response (Bouchard et al., 1999). The aforementioned studies of marathon runners showed a 4–18% increase in peak VO_2_ (Arbab-Zadeh et al., 2014; Zilinski et al., 2015), where the training was supervised and exercise doses were greater than in our study. In addition to volume, intensity of training also affects cardiorespiratory fitness response, with several studies demonstrating superiority of high intensity interval training over moderate intensity continuous training (Wisløff et al., 2007; Milanović et al., 2015; Williams et al., 2019). Therefore, training administered by experienced coaches under supervision plays an important contribution to increasing peak VO_2_, which real-world novice marathon runners following beginners training plans generally do not have. In addition, we did not observe changes in body fat, weight, or resting heart rate, which can be surrogate markers of athletic conditioning (Liou et al., 2016; Viana et al., 2019). Although we found no differences in cardiovascular remodeling or training volumes between likely cardiorespiratory responders and likely adverse responders, owing to the small number of subjects satisfying these definitions and fewer still recording training logs, there is a risk of type II statistical error, being unable to reject a false null hypothesis.

Despite an increase in peak VO_2_ not being demonstrated in this study, a small substudy of this work previously reported that muscle VO_2_, measured by near-infrared spectroscopy, increased by 48% after a first marathon run (Jones et al., 2017). Intriguingly, this suggests that adaptations in skeletal muscle improving metabolic capacity occur independently of peak VO_2_ and when cardiovascular remodeling responses are modest. Future work exploring muscle arteriolar recruitment, perfusion, and their relationships with cardiovascular afterload and BP reduction would be valuable to understand what influence exercise may have on these mechanisms.

### Myocardial Injury

By evaluating runners 16 ± 4 days after the race, we could not reproduce evidence of ventricular dysfunction or myocardial edema found in previous studies (Neilan et al., 2006a; La Gerche et al., 2012; Gaudreault et al., 2013). These studies conducted tests immediately after (Neilan et al., 2006a; La Gerche et al., 2012) or within 2 days of race completion (Gaudreault et al., 2013). We intended to avoid immediate post-race evaluation due to the inherent differences in loading conditions, circulating catecholamines, sympathetic and vasomotor activation affecting outcomes of interest. If alterations in myocardial edema, strain, systolic or diastolic function occurred in the study participants, based on these previous studies, we would expect that they should normalize by the time of our assessment (Neilan et al., 2006b; La Gerche et al., 2012; Gaudreault et al., 2013).

As transient cardiac biomarker elevation has been shown to normalize by 36 h of race completion (Shave et al., 2007, 2010; Lippi et al., 2011; Scherr et al., 2011) and we found no clinical reason to suspect persistent elevation, such as myocardial fibrosis, we did not investigate this in our study.

Future research elucidating the biological mechanisms responsible for the beneficial effects of regular exercise, such as the reduction in arterial stiffness, may yield novel therapeutic strategies, ultimately aiming to harness the anti-atherosclerotic (Nocon et al., 2008), anti-obesity, anti-diabetic, anti-osteoporotic (Warburton et al., 2010), anti-cancer (Moore et al., 2016), antidepressant, and anti-dementia properties of physical activity (Hamer et al., 2014).

The chief strengths of the study were the careful and comprehensive phenotyping using state of the art cardiovascular imaging, balanced gender inclusion, and relatively large sample size for a longitudinal study of this nature with multiple tests conducted. The recruitment process, advertising to all potential subjects through the race organizers and the inclusion of all subjects regardless of adherence to a beginner’s training plan allowed for the greatest generalizability, providing important real-world evidence on the effects of modest training on young, novice marathon runners and their cardiovascular health.

### Limitations

Although inclusivity of undertrained marathon completers can be viewed as a strength of the study, it simultaneously represents a significant weakness as detailed training information was missing from the majority of subjects, which would have been valuable in examining undertraining and further associations between training volume or intensity and cardiovascular remodeling responses. Similarly, reductions to the final sample size through loss to follow up also impaired our ability to detect small changes with accuracy.

Treadmill cardiopulmonary exercise testing would have been the preferred method to assess cardiorespiratory fitness changes in marathon runners; however, we used semi-recumbent tilting cycle ergometers to facilitate dynamic assessment of cardiac function and peripheral blood flow (Jones et al., 2017). As the post marathon cardiopulmonary exercise test took place after 7–21 days of detraining, peak VO_2_ and ventilatory threshold values may have declined from peak performance levels by variable amounts. In addition, subjects who did not achieve the recommended preparatory training may have suffered a reduction in performance after running the marathon resulting from an overreaching syndrome (Kasikcioglu et al., 2008). These limitations may have been addressed by additional assessments at interim time points during training, which would have the potential to enhance our understanding of phasic cardiac remodeling (Arbab-Zadeh et al., 2014; Weiner et al., 2015) and peak VO_2_ dynamics pre and post race.

This study did not include a control group, instead each subject acted as their own control investigating the association between training as a transient exposure and cardiovascular remodeling as an outcome. Therefore, the changes observed may have resulted from confounding factors, such as seasonal differences between October 2015 (baseline) and May 2016 (post marathon), rather than a causal effect of exercise training. BP is susceptible to seasonal differences (Alpérovitch et al., 2009), with 35 years olds experiencing a 2/2 mmHg lower BP on a warm summer day compared to a cold winter day (Brennan et al., 1982). A proposed mechanistic explanation suggests that longer daytime length and higher vitamin D levels may be responsible for a small BP reduction (Witham et al., 2009); however, exposure to sunlight is a challenging variable to control for between exercisers and sedentary controls. Though we were able to record that nine women (28%) were using combined oral contraception, one woman was using the progesterone-only pill (3%) and one woman had a levonorgestrel-releasing intrauterine system (3%) during the study, we did not obtain information regarding menstrual cycle stage at the time of testing. Oral contraceptive use can be associated with increases in BP and stages of the menstrual cycle can affect baroreflex control of sympathetic activity (Joyner et al., 2016). We were not able to evaluate these interactions in this study, which may have confounded the results.

With respect to harm in endurance running, this study was not designed to address rare but clinically important events such as sudden cardiac arrest or its causes. The majority of sports-related sudden cardiac arrests in the general population occur in men over 35 years of age with occult atherosclerotic coronary disease (Marijon et al., 2011, 2015), who were not included in this study. Future studies investigating the causes of sudden cardiac arrest during mass participation events will require a national registry with mandatory reporting (Maron et al., 2009) combined with systematic clinical investigation of victims and potentially including their families where no cause is found (Behr et al., 2008; Papadakis et al., 2013, 2018; Basso et al., 2017; Lahrouchi et al., 2017).

## Conclusion

Despite ongoing concerns regarding the cardiovascular safety of marathon running, this study demonstrates a reduction in BP and vascular stiffness in real-world, young, normotensive men and women. These benefits come despite more modest cardiovascular remodeling and cardiorespiratory fitness responses than previously reported in studies involving supervised training. We found no evidence of myocardial injury in first time marathon runners achieving an average finishing time. In clinical practice, real-world evidence of the effects of typical marathon training on cardiovascular health provides important information for the public and medical profession about an increasingly popular mass participation event.

## Data Availability Statement

The datasets generated for this study are available on request to the corresponding author.

## Ethics Statement

The studies involving human participants were reviewed and approved by the National Research Ethics Service; Queen Square, London committee granted ethical approval (15/LO/086). The participants provided their written informed consent to participate in this study.

## Author Contributions

AD drafted the manuscript, contributed to the conception and design of the work, and contributed to the acquisition, analysis, or interpretation of data for the work. JM and SS contributed to the conception or design of the work and critically revised the manuscript. AB, JZ, RB, AA-G, SJ, NN, KM, YY, JA, TT, SR, MR, PS, JW, and DC contributed to the acquisition, analysis, or interpretation of data for the work. CT, GF, EP, HD, IC, AH, RS, CM, and GL critically revised the manuscript. All authors gave final approval and agreed to be accountable for all aspects of work ensuring integrity and accuracy.

## Conflict of Interest

The authors declare that the research was conducted in the absence of any commercial or financial relationships that could be construed as a potential conflict of interest.
