# Supplementary material for: Cardiovascular Remodeling Experienced by Real-World, Unsupervised, Young Novice Marathon Runners
Source: Front Physiol. 2020 Mar 18;11:232. doi: 10.3389/fphys.2020.00232 (PMC7093496; doi:10.3389/fphys.2020.00232)
Supplement: Supplementary file 1 [file Data_Sheet_1.PDF]

Beginner 17 Week Training Plan

Introduction: The following training programmes cover a period of 17 weeks. Before embarking on one of these marathon training plans you should have done four to eight weeks of steady running so that you have a foundation of fitness to build on. Each programme is designed to cater for different levels and abilities so it's important that you choose the right plan for you. A training schedule needs to be challenging but not so that you feel out of your depth.

Key: ER = Easy Run, SR = Steady Run, TR = Threshold Run, HR = Hill Run, FR = Fartlek Run, IR = Interval Run, LR = Long Run, MP = Marathon Pace, HMP = Half Marathon Pace, H&N = Hydration & Nutrition Strategies

**Week 1**	
**Monday**	Rest
**Tuesday**	10min walk, 20min ER, 5min walk
**Wednesday**	Rest
**Thursday**	10min walk, 30min ER, 5min walk
**Friday**	Core & Stretching
**Sat or Sun**	5min walk, 30min LR, 5min walk, 10min ER, 5min walk

**Week 2**	
**Monday**	Rest
**Tuesday**	35min ER
**Wednesday**	Rest
**Thursday**	5min walk, 35min ER, 5min walk
**Friday**	Core & Stretching
**Sat or Sun**	5min walk, 45min LR, 10min walk

Beginner 17 Week Training Plan

Week 3	
**Monday**	Rest
**Tuesday**	40min ER
**Wednesday**	Rest
**Thursday**	15min ER, 10min SR, 15min ER
**Friday**	Core & Stretching
**Sat or Sun**	5min walk, 55min LR, 5min walk

Week 4	
**Monday**	Rest
**Tuesday**	45min ER
**Wednesday**	Rest
**Thursday**	45min FR as 5min ER, 10min SR, 5min ER, 5min TR, 10min ER, 5min SR, 5min ER
**Friday**	Core & Stretching
**Sat or Sun**	5min walk, 65min LR, 5min walk

Week 5	
**Monday**	Rest
**Tuesday**	45min ER
**Wednesday**	Rest
**Thursday**	45min FR as 5min ER, 15min SR, 5min ER, 5min TR, 5min ER, 5min SR, 5min ER
**Friday**	Core & Stretching
**Sat or Sun**	5min walk, 75min LR, 5min walk

Beginner 17 Week Training Plan

Week 6	
**Monday**	Rest
**Tuesday**	10min ER, 5 x (3min IR, 2min ER), 15min ER
**Wednesday**	Rest
**Thursday**	45min FR as 10min ER, 15min SR, 5min ER, 10min TR, 5min ER
**Friday**	Core & Stretching
**Sat or Sun**	10 miles LR

Week 7	(An easier week to help your body recover and adapt to the training)
**Monday**	Rest
**Tuesday**	20min ER
**Wednesday**	Rest
**Thursday**	40min ER
**Friday**	Core & Stretching
**Sat or Sun**	50min ER

Week 8	
**Monday**	Rest
**Tuesday**	10min ER, 8 x (2min IR, 2min ER), 10min SR
**Wednesday**	Rest
**Thursday**	10min ER, 3 x (7min TR, 2min ER), 10min ER
**Friday**	Core & Stretching
**Sat or Sun**	12 miles LR. Practise H&N

Beginner 17 Week Training Plan

Week 9	
**Monday**	Rest
**Tuesday**	40min ER
**Wednesday**	Rest
**Thursday**	50min SR
**Friday**	Core & Stretching
**Sat or Sun**	14 miles LR. Practise H&N

Week 10	
**Monday**	Rest
**Tuesday**	50min ER
**Wednesday**	Rest
**Thursday**	10min ER, 20min HR, 5min ER, 10min SR
**Friday**	Core & Stretching
**Sat or Sun**	16 miles LR. Practise H&N

Week 11	
**Monday**	Rest
**Tuesday**	10min ER, 5 x (5min IR, 2.5min ER), 10min ER
**Wednesday**	Rest
**Thursday**	40min ER
**Friday**	Core & Stretching
**Sat or Sun**	Run a Half Marathon<br>https://www.adidashalfmarathon.com/onlinentry/register/1/

Beginner 17 Week Training Plan

Week 12	
**Monday**	Rest
**Tuesday**	50min ER
**Wednesday**	Rest
**Thursday**	10min ER, 3 x (8min TR, 2min ER), 5 x 30sec fast, 5min ER
**Friday**	Core & Stretching
**Sat or Sun**	18 miles LR 3 x 4 miles MP at start, middle and end. Practise H&N

Week 13	
**Monday**	Rest
**Tuesday**	35min ER
**Wednesday**	Rest
**Thursday**	5min ER, 40min SR, 5min ER
**Friday**	Core & Stretching
**Sat or Sun**	20 miles LR. Practise H&N

4 weeks to go!	
**Monday**	Rest
**Tuesday**	30min ER
**Wednesday**	Rest
**Thursday**	1 mile ER, 4 miles HMP, 1 mile ER
**Friday**	Core & Stretching
**Sat or Sun**	22 miles LR. This will be your final long training run. Practise MP and H&N

Beginner 17 Week Training Plan

3 weeks to go!	
**Monday**	Rest
**Tuesday**	30 min ER
**Wednesday**	Rest
**Thursday**	2 miles ER, 4 miles HMP, 2 miles ER
**Friday**	Core & Stretching
**Sat or Sun**	13 miles LR. Practise MP and H&N

2 weeks to go!	
**Monday**	Rest
**Tuesday**	30 min ER
**Wednesday**	Rest
**Thursday**	1 mile ER, 5 x (2 mins IR, 2 mins ER), 1 mile ER
**Friday**	Core & Stretching
**Saturday**	8 miles ER

1 week to go!	
**Monday**	10 mins MP, 20 mins HMP, 10 mins MP
**Tuesday**	Rest
**Wednesday**	20min ER
**Thursday**	Rest
**Friday**	20min ER
**Saturday**	Gentle Stretching
**Sunday**	Race day! Remember to stretch and warm down with a 15 min walk. Eat and drink well.
