# Supplementary material for: Cardiovascular Remodeling Experienced by Real-World, Unsupervised, Young Novice Marathon Runners
Source: Front Physiol. 2020 Mar 18;11:232. doi: 10.3389/fphys.2020.00232 (PMC7093496; doi:10.3389/fphys.2020.00232)
Supplement: Supplementary file 2 [file Data_Sheet_2.docx]

Supplementary Material

# Supplementary Data

**Table of Contents**

| **Tables and Figures** |  |
| --- | --- |
| S1 Table. Ergometer ramp protocol based on gender and height. | 2 |
| S2 Table. Cardiac imaging, haemodynamic, cardiorespiratory and allometric measurements of the injured cohort at baseline and post marathon. These subjects did not run the marathon but returned for a second evaluation after deferring their marathon places. | 3 |
| S3 Table. Training data returned by 38 subjects. | 5 |
| S4 Table. Additional cardiac imaging, haemodynamic, cardiorespiratory, electrocardiographic, haematological and biochemical measurements at baseline and post marathon. | 7 |
| S5 Table. Additional cardiac imaging, cardiorespiratory and electrocardiographic measurements at baseline and post marathon, separated by gender. | 12 |
| S6 Table. Comparison of the cardiac imaging, haemodynamic, peak VO_2_ measurements and training volumes of the likely cardiorespiratory responders and likely adverse responders. | 14 |
| S7 Table. Inter-observer intraclass correlation coefficients for cardiovascular magnetic resonance imaging indices. | 16 |
| S8 Table. Intra-observer intraclass correlation coefficients for cardiovascular magnetic resonance imaging indices repeated analysis 6 months apart by the same observer. | 17 |
| S1 Figure. Weekly distances run by 38 subjects returning training logs. | 18 |
| S2 Figure. Weekly running pace by 38 subjects returning training logs. | 19 |

## S1 Table. Ergometer ramp protocol based on gender and height.

|  | Male |  | Female |
| --- | --- | --- | --- |
| Height <170cm | 20 W | Height <160cm | 15 W |
| 170cm ≤ Height ≤190cm | 25 W | 176cm ≤ Height ≤180cm | 20 W |
| Height >190cm | 30 W | Height >180cm | 25 W |

W, Watts per minute increments selected.

All subjects had 2 minutes of resting data recorded on the cycle ergometer, followed by 3 minutes of unloaded cycling in a familiarization warm-up phase. Subjects were advised to pedal at a speed of 60 – 70 revolutions/min, at a comfortable cadence. The cardiopulmonary exercise test was then commenced with the incremental ramp protocol described above. When subjects exercised maximally to volitional exhaustion, recovery data was collected for 5 minutes, ensuring heart rate and blood pressure returned to baseline values.

## S2 Table. Cardiac imaging, haemodynamic, cardiorespiratory and allometric measurements of the injured cohort at baseline and post marathon. These subjects did not run the marathon but returned for a second evaluation after deferring their marathon places.

|  | Baseline | Post marathon | *P* value |
| --- | --- | --- | --- |
| CMR and haematocrit |  |  |  |
| iLV EDV (ml/m^2^) | 90.0 ±12.8 | 87.5 ±11.1 | 0.12 |
| iLV ESV (ml/m^2^) | 33.4 ±5.7 | 33.0 ±5.9 | 0.60 |
| LV EF (%) | 62.7 ±5.5 | 62.4 ±5.2 | 0.73 |
| iLV mass (g/m^2^) | 63.4 ±9.8 | 63.6 ±9.1 | 0.80 |
| Mean LV wall thickness (mm) | 6.8 ±0.8 | 7.0 ±0.8 | 0.04 |
| Hematocrit | 0.41 ±0.04 | 0.43 ±0.04 | <0.01 |
| iRV EDV (ml/m^2^) | 93.0 ±15.4 | 89.4 ±13.6 | 0.06 |
| iRV ESV (ml/m^2^) | 41.5 ±7.2 | 41.0 ±7.9 | 0.61 |
| RV EF (%) | 55.4 ±4.4 | 54.4 ±3.7 | 0.21 |
| CPET** |  |  |  |
| Peak VO_2_ (ml/min/kg) | 37.0 ±6.2 | 36.9 ±6.3 | 0.89 |
| Percentage predicted peak VO_2_ (%) | 109.6 ±15.5 | 109.2 ±14.5 | 0.90 |
| Ventilatory threshold as percentage of peak VO_2_ (%) | 57.9 ±13.0 | 57.1 ±9.4 | 0.80 |
| Exercise time (secs) | 588.0 ±109.0 | 612.8 ±82.9 | 0.15 |
| Peak power (Watt) | 218 ±61 | 228 ±53 | 0.02 |
| OUES (ml/min/L/min) | 2517 [2352, 3107] | 2480 [2142, 2968] | 0.14 |
| Peak HR (bt/min) | 170 ±19 | 174 ±16 | 0.32 |
| Peak HR percentage predicted (%) | 88.6 ±9.6 | 91.3 ±8.1 | 0.25 |
| Peak RER | 1.21 ±0.1 | 1.23 ±0.1 | 0.33 |
| Blood pressure |  |  |  |
| Systolic BP (mmHg) | 118 ±11 | 117 ±9 | 0.50 |
| Diastolic BP (mmHg) | 76 ±6 | 73 ±6 | 0.39 |
| CMR whole aorta PWV (m/s) | 5.1 ±0.6 | 5.1 ±0.9 | 0.80 |
| Allometry and renal function |  |  |  |
| Body Mass Index | 24.0 ±3.1 | 24.1 ±3.3 | 0.47 |
| Body fat (%) | 24.6 ±8.0 | 25.4 ±7.3 | 0.07 |
| Creatinine (μmol/L) | 71 ±11 | 71 ±13 | 0.91 |

Data expressed as mean ±SD if normally distributed. If non-normally distributed data expressed as median [IQR].

BP, blood pressure; CMR, cardiac magnetic resonance; CPET, cardiopulmonary exercise test; ECV, extracellular volume; EDV, end-diastolic volume; EF, ejection fraction; ESV, end-systolic volume; HR, heart rate; iLV indexed left ventricular; iRV, indexed right ventricular; LV, left ventricular; max, maximal; OUES, oxygen uptake efficiency slope; PWV, pulse wave velocity; RER, respiratory exchange ratio; RV, right ventricular; VO_2_ oxygen consumption.

** 4 subjects who returned for testing but with injuries did not perform CPET

## S3 Table. Training data returned by 38 subjects.

| Week | Median distance run (km) | Median time spent exercising (HH:MM:SS) | Mean pace (min/km) | Training plan exercise time | Proportion of training plan (%) |
| --- | --- | --- | --- | --- | --- |
| 1 | 4.1 (0, 12.6) | 00:35:24 (00:00:00, 01:13:04) | 6.0 ±0.9 |  |  |
| 2 | 1.6 (0, 11.2) | 00:25:12 (00:00:00, 01:15:19) | 5.8 ±1.1 |  |  |
| 3 | 5.0 (0, 10.0) | 00:29:30 (00:00:00, 01:06:16) | 5.8 ±0.9 |  |  |
| 4 | 2.6 (0, 12.7) | 00:36:34 (00:00:00, 01:10:49) | 5.6 ±0.5 |  |  |
| 5 | 3.2 (0,15.6) | 00:21:41 (00:00:00, 01:31:21) | 6.0 ±0.8 |  |  |
| 6 | 6.2 (0, 19.5) | 00:50:06 (00:00:00, 01:57:05) | 5.8 ±0.7 |  |  |
| 7 | 8.3 (0, 16.4) | 00:52:30 (00:00:00, 01:46:25) | 5.8 ±0.8 |  |  |
| 8 | 3.6 (0, 11.1) | 00:21:29 (00:00:00, 01:24:14) | 5.6 ±0.6 |  |  |
| 9 | 1.8 (0, 13.5) | 00:10:20 (00:00:00, 01:22:30) | 5.8 ±0.7 |  |  |
| 10 | 0 (0, 15.8) | 00:00:00 (00:00:00, 01:48:10) | 5.9 ±1.0 |  |  |
| 11 | 0.9 (0, 7.8) | 00:05:00 (00:00:00, 00:58:41) | 5.8 ±0.9 |  |  |
| 12 | 5.0 (0, 12.3) | 00:34:41 (00:00:00, 01:10:56) | 5.9 ±1.1 |  |  |
| 13 | 10.2 (0, 25.2) | 01:08:30 (00:00:00, 02:20:00) | 5.8 ±0.9 | 01:30:00 | 76 |
| 14 | 15.9 (4.4, 29.5) | 01:49:54 (00:29:10, 03:08:50) | 5.7 ±0.7 | 01:55:00 | 96 |
| 15 | 18.8 (2.6, 32.4) | 01:47:42 (00:14:50, 03:06:30) | 5.6 ±0.6 | 02:15:00 | 80 |
| 16 | 18.6 (0.3, 29.1) | 01:43:58 (00:02:00, 02:53:40) | 5.8 ±0.7 | 02:35:00 | 67 |
| 17 | 20.9 (8.3, 35.5) | 02:25:06 (00:44:50, 03:31:10) | 5.6 ±0.6 | 02:45:00 | 88 |
| 18 | 26.1 (6.8, 40.0) | 02:20:48 (01:12:30, 03:42:00) | 5.6 ±0.7 | 03:00:00 | 78 |
| 19 | 28.2 (13.3, 40.2) | 02:31:12 (01:24:24, 04:01:20) | 5.6 ±0.6 | 01:50:00 | 137 |
| 20 | 28.3 (16.1, 36.3) | 02:53:30 (01:37:19, 03:53:20) | 5.7 ±0.7 | 03:29:00 | 83 |
| 21 | 28.5 (14.8, 40.9) | 03:01:10 (01:13:46, 03:52:20) | 5.7 ±0.8 | 03:40:00 | 82 |
| 22 | 24.0 (8.3, 36.4) | 02:13:42 (00:47:09, 04:01:00) | 5.9 ±0.9 | 04:05:00 | 55 |
| 23 | 29.4 (6.6, 39.3) | 02:46:16 (00:43:25, 04:06:50) | 5.7 ±0.8 | 03:40:00 | 76 |
| 24 | 33.0 (0, 46.2) | 03:15:40 (00:00:00, 04:35:10) | 5.7 ±0.6 | 04:30:00 | 72 |
| 25 | 20.6 (1.9, 42.1) | 01:53:06 (00:14:59, 04:22:00) | 5.8 ±0.9 | 04:35:00 | 41 |
| 26 | 29.2 (15.5, 51.0) | 03:03:40 (01:41:04, 04:58:00) | 5.9 ±0.8 | 04:50:00 | 63 |
| 27 | 26.3 (8.7, 39.1) | 02:40:44 (00:53:07, 03:57:20) | 5.8 ±0.7 | 03:40:00 | 73 |
| 28 | 14.3 (0, 27.0) | 01:41:27 (00:00:00, 02:37:20) | 5.6 ±0.7 | 02:00:00 | 85 |
| 29 | 43.7 (42.2, 49.4) | 04:32:00 (03:43:40, 05:24:20) | 6.1 ±0.9 | 01:20:00 | Race week |
| 30 | 0 (0, 4.7) | 00:00:00 (00:00:00, 00:26:15) | 5.2 ±0.9 |  |  |
| 31 | 0 (0, 7.9) | 00:10:01 (00:00:00, 00:53:03) | 5.4 ±0.8 |  |  |
| 32 | 0 (0, 1.4) | 00:00:00 (00:00:00, 00:18:05) | 5.4 ±0.7 |  |  |
| 33 | 0 (0, 7.4) | 00:00:00 (00:00:00, 01:16:53) | 5.7 ±0.7 |  |  |
| 34 | 0 (0, 6.5) | 00:00:00 (00:00:00, 00:55:21) | 5.8 ±1.2 |  |  |
| 35 | 0 (0, 5.9) | 00:00:00 (00:00:00, 00:42:54) | 6.1 ±1.0 |  |  |
| 36 | 0 (0, 6.8) | 00:09:22 (00:00:00, 01:04:01) | 5.6 ±0.7 |  |  |
| 37 | 0 (0, 0.4) | 00:00:00 (00:00:00, 00:41:32) | 5.5 ±0.6 |  |  |
| 38 | 0 (0, 1.9) | 00:00:00 (00:00:00, 00:42:10) | 5.7 ±0.8 |  |  |
| 39 | 0 (0, 0) | 00:00:00 (00:00:00, 00:38:57) | 5.8 ±1.0 |  |  |
| 40 | 0 (0, 3.6) | 00:00:00 (00:00:00, 00:41:00) | 5.6 ±0.6 |  |  |
| 41 | 0 (0, 1.7) | 00:00:00 (00:00:00, 00:37:26) | 5.6 ±0.7 |  |  |
| 42 | 0 (0, 5.8) | 00:00:00 (00:00:00, 01:06:30) | 5.7 ±0.9 |  |  |
| 43 | 0 (0, 2.5) | 00:00:00 (00:00:00, 00:33:45) | 5.7 ±1.0 |  |  |

## S4 Table. Additional cardiac imaging, haemodynamic, cardiorespiratory, electrocardiographic, haematological and biochemical measurements at baseline and post marathon.

|  | Baseline | Post marathon | *P* value |
| --- | --- | --- | --- |
| Echocardiography |  |  |  |
| IVS (cm) | 0.72 ±0.14 | 0.75 ±0.14 | 0.02 |
| PW (cm) | 0.73 ±0.11 | 0.76 ±0.15 | 0.18 |
| Mean LV wall thickness (cm) | 0.72 ±0.11 | 0.75 ±0.13 | 0.02 |
| LV Internal Dimension (mm) | 47.4 ±5.2 | 48.5 ±4.8 | 0.01 |
| LV length (mm) | 85.5 ±8.2 | 85.7 ±7.7 | 0.76 |
| EDV (ml) | 108.8 ±29.7 | 116.6 ±33.9 | <0.01 |
| ESV (ml) | 45.9 ±14.2 | 48.7 ±16.6 | 0.03 |
| E wave (m/s) | 0.78 ±0.17 | 0.76 ±0.15 | 0.33 |
| A wave (m/s) | 0.49 ±0.10 | 0.48 ±0.10 | 0.16 |
| Lateral e’ (cm/s) | 18.28 ±3.46 | 19.18 ±3.31 | 0.06 |
| Lateral a’ (cm/s) | 7.82 ± 1.91 | 8.15 ±1.78 | 0.20 |
| Lateral s’ (cm/s) | 12 [10, 14] | 11 [10, 13] | 0.10 |
| Medial e’ (cm/s) | 12.63 ±2.71 | 13.42 ±3.04 | 0.09 |
| Medial a’ (cm/s) | 7.6 ±1.90 | 7.85 ±1.86 | 0.34 |
| Medial s’ (cm/s) | 8 [7,9] | 9 [8, 10] | 0.15 |
| E/A ratio | 1.47 [1.33, 1.90] | 1.52 [1.34, 1.95] | 0.38 |
| RV TAPSE (cm) | 2.46 ±0.41 | 2.47 ±0.40 | 0.82 |
| RV e’ (cm/s) | 14.30 ±3.41 | 13.97 ±2.91 | 0.69 |
| RV a’ (cm/s) | 11.96 ±4.12 | 11.71 ±3.53 | 0.21 |
| RV s’ (cm/s) | 13.9 ±2.56 | 13.03 ±2.07 | <0.01 |
| Global longitudinal strain % | -18.0 ±2.8 | -18.7 ±2.1 | 0.06 |
| RV free wall LS | -22.0 [-24.5, -17.0] | -21.3 [-25.0, -14.7] | 0.54 |
| Peak rotation Mitral Valve level (^o^) | -2.1 ±6.1 | -3.1 ±6.0 | 0.20 |
| Peak rotation Papillary Muscle level (^o^) | 1.9 ±5.2 | 1.2 ±4.3 | 0.27 |
| Peak rotation Apical level (^o^) | 8.2 ±4.9 | 9.1 ±6.5 | 0.30 |
| LV Twist (^o^) | 10.1 ±6.9 | 12.0 ±8.2 | 0.13 |
| LV Torsion (^o^/cm) | 1.2 ±0.8 | 1.4 ±0.9 | 0.17 |
| CMR |  |  |  |
| LV EDV (ml) | 170.3 ±38.6 | 175.3 ±39.9 | <0.01 |
| LV ESV (ml) | 62.33 ±17.89 | 64.77 ±18.99 | 0.02 |
| LV SV (ml) | 108.0 ±24.8 | 110.6 ±25.3 | 0.14 |
| iLV SV (ml/m^2^) | 57.8 ±9.6 | 59.5 ±9.8 | <0.01 |
| LV mass (g) | 122.4 ±32.1 | 127.2 ±31.4 | <0.01 |
| Mass: Volume (g/ml) | 0.72 ±0.08 | 0.73 ±0.08 | 0.22 |
| Myocardial partition coefficient λ (%) | 44.7 ±3.2 | 45.2 ±3.1 | 0.32 |
| T1 myocardium post contrast | 630 ±40.76 | 638 ±35.29 | 0.16 |
| T1 blood | 1601 ±69.07 | 1608 ±89.29 | 0.44 |
| T1 blood post contrast | 517 ±52.62 | 531 ±51.37 | 0.11 |
| RV EDV (ml) | 173.2 ±38.6 | 180.6 ±41.9 | <0.01 |
| RV ESV (ml) | 75.2 ±18.8 | 78.2 ±21.1 | 0.01 |
| RV SV (ml) | 98.1 ±22.7 | 102.4 ±23.7 | 0.02 |
| iRV SV (ml/m^2^) | 52.5 ±8.8 | 55.0 ±9.0 | <0.01 |
| LA Biplanar volume (ml) | 66.9 ±22.2 | 70.4 ±21.5 | 0.06 |
| LA max 4ch volume (ml) | 70.6 ±20.9 | 73.9 ±20.7 | 0.15 |
| iLA max 4ch volume (ml/m^2^) | 38.0 ±10.1 | 39.9 ±10.0 | 0.10 |
| RA max 4ch volume (ml) | 82.5 ±23.7 | 87.9 ±25.5 | <0.01 |
| CMR Global longitudinal strain % | -21.8 ±2.3 | -21.5 ±2.2 | 0.31 |
| CMR Peak basal LV rotation (^o^) | -9.4 [-12.4, -5.6] | -8.7 [-11.6, -5.6] | 0.96 |
| CMR Peak apical LV rotation (^o^) | 4.6 [-6.7, 9.0] | 4.6 [-6.0, 8.2] | 0.55 |
| CMR LV Twist (^o^) | 8.2 ±12.9 | 9.1 ±11.9 | 0.66 |
| CPET |  |  |  |
| Peak VO_2_ (ml/min) | 2701 ±678 | 2744 ±684 | 0.32 |
| Ventilatory threshold (ml/min/kg) | 23.2 ±5.3 | 21.8 ±4.3 | 0.02 |
| Peak minute ventilation VE (L/min) | 100 ±30 | 92 ±27 | <0.01 |
| Peak tidal volume VT (L) | 2.36 [1.78, 2.75] | 2.23 [1.83, 2.87] | 0.49 |
| Peak respiratory frequency Rf (1/min) | 43.0 ±11.6 | 40.0 ±8.9 | 0.06 |
| Peak HR (bpm) | 170 [162, 178] | 171 [160, 187] | 0.31 |
| Peak HR % predicted | 88.7 ±7.9 | 87.6 ±6.3 | 0.37 |
| VO_2_/WR slope (ml/min/Watt) | 9.7 ±0.9 | 10.1 ±0.8 | 0.02 |
| Peak VE/VO_2_ | 37.2 ±6.4 | 35.8 ±4.9 | 0.08 |
| Peak VE/VCO_2_ | 31.4 ±5.1 | 30.3 ±3.8 | 0.08 |
| Electrocardiography |  |  |  |
| Resting heart rate (bpm) | 66 ±14 | 64 ±13 | 0.31 |
| PR interval (ms) | 149 [134, 167] | 152 [138, 171] | 0.56 |
| QRS duration (ms) | 91 ±10 | 91 ± 10 | 0.59 |
| QTc interval (ms) | 415 ±27 | 412 ±28 | 0.37 |
| Sokolow-Lyon LV voltage (mm) | 24.7 ±0.6 | 25.1 ±0.7 | 0.41 |
| LV hypertrophy (%) | 5.9 | 10.3 | 0.25 |
| RV hypertrophy (%) | 0 | 0 | NA |
| First-degree AVB block (%) | 4.4 | 5.9 | NS |
| Any early repolarisation pattern (%) | 7.4 | 7.4 | NS |
| Blood tests |  |  |  |
| Haemoglobin (g/dL) | 140 ±12.4 | 143 ±14 | 0.11 |
| White cell count (x10*9/L) | 6.3 ±1.8 | 6.3 ±1.7 | 0.75 |
| Platelets (x10*9/L) | 255 ±67 | 264 ±65 | 0.30 |
| Sodium (mmol/L) | 141 ±1.5 | 140 ±2.0 | 0.14 |
| Potassium (mmol/L) | 4.2 ±0.3 | 4.2 ±0.3 | 0.35 |
| Urea (mmol/L) | 4.8 ±1.1 | 5.0 ±1.1 | 0.08 |

Data expressed as mean ±SD if normally distributed. If non-normally distributed data expressed as median [IQR].

4ch, four-chamber; AVB, Atrio-ventricular block; CMR, cardiac magnetic resonance; CPET, cardiopulmonary exercise test; EDV, end-diastolic volume; ESV, end-systolic volume; HR, heart rate; iLA, indexed left atrial; iLV indexed left ventricular; iRA, indexed right atrial; iRV, indexed right ventricular; IVS, interventricular septum; LA, left atrial; LS, longitudinal strain; LV, left ventricular; max, maximal; NA, not available; NS, not significant, *P* value >0.99; PW, posterior wall; PWV, pulse wave velocity; RV, right ventricular; SV, stroke volume; VE, minute ventilation; VCO_2_, carbon dioxide output; VO_2,_ oxygen consumption; WR, work rate.

## S5 Table. Additional cardiac imaging, cardiorespiratory and electrocardiographic measurements at baseline and post marathon, separated by gender.

|  | **Baseline**  **male subjects** | **Post marathon**  **male subjects** | **Change** | ***P* value** | **Baseline**  **female subjects** | **Post marathon female subjects** | **Change** | ***P* value** |
| --- | --- | --- | --- | --- | --- | --- | --- | --- |
| LV EDV (ml) | 195.5 ±34.0 | 200.1 ±36.0 | 5 | 0.07 | 141 ± 16.7 | 146.6 ±20.0 | 6 | <0.01 |
| LV ESV (ml) | 72.1 ±18.5 | 75.6 ±18.5 | 4 | 0.02 | 51.0 ±7.6 | 52.2 ±9.6 | 1 | 0.42 |
| LV SV (ml) | 123.4 ±21.1 | 124.5 ±23.5 | 1 | 0.66 | 90.1 ±14.6 | 94.4 ±16.0 | 4 | 0.07 |
| iLV SV (ml/m^2^) | 61.8 ±8.3 | 62.8 ±9.3 | 1 | 0.48 | 53.2 ±9.0 | 55.7 ±9.0 | 3 | 0.06 |
| LV mass (g) | 145.4 ±25.6 | 150.5 ±23.1 | 5 | <0.01 | 95.6 ±11.2 | 100.1 ±11.9 | 5 | <0.01 |
| Mass: Volume (g/ml) | 0.75 ±0.1 | 0.76 ±0.1 | 0.01 | 0.21 | 0.68 ±0.1 | 0.69 ±0.1 | 0.01 | 0.52 |
| RV EDV (ml) | 199.4 ±32.3 | 206.8 ±37.2 | 7 | 0.04 | 142.8 ±16.6 | 150.1 ±21.5 | 7 | <0.01 |
| RV ESV (ml) | 87.4 ±16.0 | 90.7 ±19.3 | 3 | 0.08 | 61.0 ±9.6 | 63.7 ±11.8 | 3 | 0.08 |
| RV SV (ml) | 112.1 ±20.4 | 116.1 ±22.3 | 4 | 0.15 | 81.8 ±11.9 | 86.5 ±12.7 | 5 | 0.04 |
| iRV SV (ml/m^2^) | 56.3 ±8.9 | 58.5 ±9.1 | 2 | 0.11 | 48.2 ±6.4 | 51.0 ±7.0 | 3 | 0.04 |
| LA Biplanar volume (ml) | 75.6 ±22.5 | 79.2 ±20.3 | 4 | 0.21 | 56.7 ± 17.3 | 60.2 ± 18.4 | 4 | 0.15 |
| RA max 4ch volume (ml) | 95.7 ±20.8 | 102.0 ±22.5 | 6 | 0.05 | 67.2 ±16.8 | 71.5 ±17.9 | 4 | 0.08 |
| CPET |  |  |  |  |  |  |  |  |
| Peak VE (L/min) | 119 ±26 | 109 ±24 | -10 | 0.02 | 79 ±19 | 75 ±16 | -4 | 0.12 |
| Peak tidal volume VT (L) | 2.7 [2.5, 3.0] | 2.9 [2.4, 3.1] | 0.2 | 0.68 | 1.8 [1.7, 2.0] | 1.8 [1.7, 2.1] | 0 | 0.49 |
| Peak respiratory frequency Rf (1/min) | 44 ±12 | 40 ±9 | -3 | 0.15 | 42 ±12 | 40 ±9 | -2 | 0.24 |
| Peak HR (bpm) | 171 [160, 178] | 174 [159, 188] | 3 | 0.31 | 167 [164, 177] | 174 [159, 188] | 7 | 0.45 |
| Peak HR % predicted | 89 ±8 | 88 ±7 | -1 | 0.74 | 89 ±9 | 87 ±6 | -2 | 0.38 |
| ECG |  |  |  |  |  |  |  |  |
| HR (bpm) | 65 ±14 | 64 ±15 | -1 | 0.82 | 68 ±14 | 65 ±11 | -3 | 0.18 |
| PR interval (ms) | 156 [142, 173] | 155 [144, 188] | NA | 0.46 | 147 [132, 157] | 144 [136, 160] | NA | 0.95 |
| QRS duration (ms) | 97 ±10 | 96 ±9 | -1 | 0.96 | 85 ±8 | 85 ±7 | 0 | 0.32 |
| QTc interval (ms) | 406 ±26 | 403 ±29 | -3 | 0.44 | 425 ±25 | 423 ±22 | -2 | 0.65 |

Data expressed as mean ±SD if normally distributed. If non-normally distributed data expressed as median [IQR].

BP, blood pressure; CMR, cardiac magnetic resonance; CPET, cardiopulmonary exercise test; ECG, electrocardiography; ECV, extracellular volume; EDV, end-diastolic volume; ESV, end-systolic volume; HR, heart rate; iLV indexed left ventricular; iRV, indexed right ventricular; LV, left ventricular; max, maximal; METS, metabolic equivalent of task; OUES, oxygen uptake efficiency slope; PWV, pulse wave velocity; RA, right atrial; RV, right ventricular; SV, stroke volume; VE, minute ventilation; VO_2_ oxygen consumption; WR, work rate.

## S6 Table. Comparison of the cardiac imaging, haemodynamic, peak VO_2_ measurements and training volumes of the likely cardiorespiratory responders and likely adverse responders.

|  | Likely responders (n=7) | Likely adverse responders (n=5) | *P* value |
| --- | --- | --- | --- |
| CMR |  |  |  |
| Relative change in iLV EDV (%) | +7.0 | +8.8 | 0.79 |
| Absolute change in LV EF (%) | -1.8 | +3.4 | 0.19 |
| Relative change in iLV mass (%) | +8.3 | +8.5 | 0.97 |
| Relative change in iRV EDV (%) | +12.2 | +5.2 | 0.40 |
| Absolute change in RV EF (%) | -0.1 | +0.4 | 0.86 |
| CPET |  |  |  |
| Relative change in peak VO_2_ (ml/min/kg) | +22.5 | -24.2 | <0.01 |
| Blood pressure and aortic PWV |  |  |  |
| Absolute change in systolic BP (mmHg) | -5 | -5 | 0.96 |
| Absolute change in diastolic BP (mmHg) | +1 | -3 | 0.56 |
| Relative change in CMR whole aorta PWV (%) | -7.4 | -3.5 | 0.65 |
|  | Likely responders (n=4) | Likely adverse responders (n=3) | *P* value |
| Training volume* |  |  |  |
| Mean weekly training time (s) | 4873 | 5141 | 0.76 |
| Mean weekly training distance (km) | 26.3 | 26.3 | 0.99 |
| Mean running pace (min/km) | 4.37 | 5.00 | 0.15 |

BP, blood pressure; CMR, cardiac magnetic resonance; CPET, cardiopulmonary exercise test; ECG, electrocardiography; ECV, extracellular volume; EDV, end-diastolic volume; ESV, end-systolic volume; iLV indexed left ventricular; iRV, indexed right ventricular; LV, left ventricular; PWV, pulse wave velocity; VO_2_ oxygen consumption.

*Of the likely responder group 4/7 provided training logs and of the likely adverse responder group 3/5 provided training logs. The mean of the weekly training time, distance and running pace are given for the 17-week progressive training period.

## S7 Table. Inter-observer intraclass correlation coefficients for cardiovascular magnetic resonance imaging indices.

|  | **ICC** | **Confidence interval** | ***P* value** |
| --- | --- | --- | --- |
| LV EDV (ml) | 0.87 | 0.07, 0.97 | 0.02 |
| LV EF (%) | 0.73 | 0.39, 0.90 | <0.01 |
| LV mass (g) | 0.82 | -0.01, 0.96 | 0.03 |
| RV EDV (ml) | 0.97 | 0.81, 0.99 | <0.01 |
| RV EF (%) | 0.85 | 0.30, 0.96 | <0.01 |
| Native T1 myocardium (ms) | 0.89 | 0.84, 0.93 | <0.01 |
| Native T1 blood pool (ms) | 0.99 | 0.98, 0.99 | <0.01 |
| ECV (%) | 0.75 | 0.43, 0.88 | <0.01 |

EDV, end-diastolic volume; EF, ejection fraction; ESV, end-systolic volume; ICC, intraclass correlation coefficient; LV, left ventricular; RV, right ventricular.

## S8 Table. Intra-observer intraclass correlation coefficients for cardiovascular magnetic resonance imaging indices repeated analysis 6 months apart by the same observer.

|  | **ICC** | **Confidence interval** | ***P* value** |
| --- | --- | --- | --- |
| LV EDV (ml) | 0.94 | 0.38, 0.99 | <0.01 |
| LV EF (%) | 0.81 | 0.51, 0.93 | <0.01 |
| LV mass (g) | 0.87 | 0.63, 0.96 | <0.01 |
| RV EDV (ml) | 0.99 | 0.97, 0.996 | <0.01 |
| RV EF (%) | 0.76 | 0.43, 0.91 | <0.01 |

EDV, end-diastolic volume; EF, ejection fraction; ESV, end-systolic volume; ICC, intraclass correlation coefficient; LV, left ventricular; RV, right ventricular.

## S2 Figure. Weekly distances run by 38 subjects returning training logs.

**
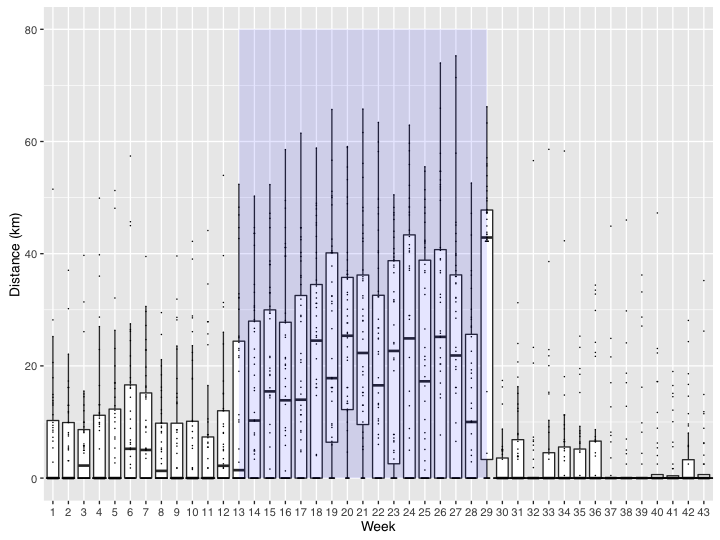
**

Shaded blue area represents the 17-Week Beginners Training Plan period. Boxplots represent the weekly distribution of running pace, highlighting the median and interquartile ranges.

## S3 Figure. Weekly running pace by 38 subjects returning training logs.
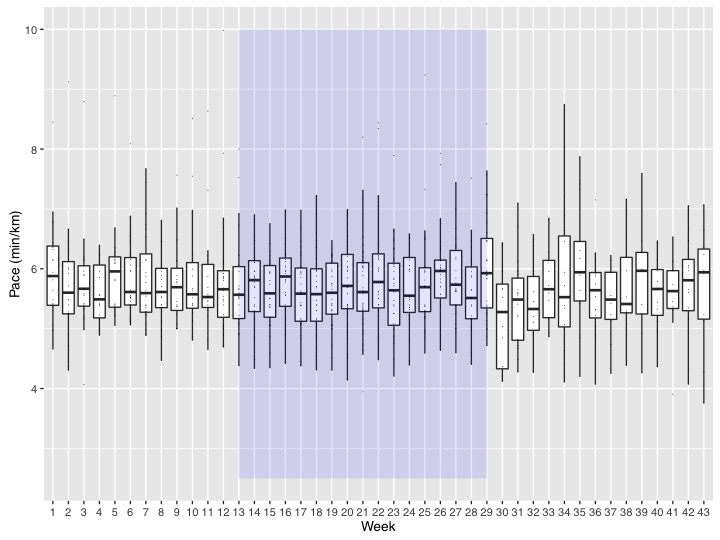


Shaded blue area represents the 17-Week Beginners Training Plan period. Boxplots represent the weekly distribution of running pace, highlighting the median and interquartile ranges.

## 
